# Enablers and obstacles for designing and implementing intersectoral health policies: Lessons from Mexico

**DOI:** 10.1002/puh2.190

**Published:** 2024-06-24

**Authors:** Adolfo Martinez‐Valle, Alejandro Figueroa‐Lara

**Affiliations:** ^1^ Population and Health Policy Research Center, Faculty of Medicine National Autonomous University of Mexico Mexico City Mexico; ^2^ San Diego California USA

**Keywords:** health policy process, intersectoral governance, Mexico, social determinants

## Abstract

**Background:**

This study analyzes how national intersectoral public policy experiences have been adopted and implemented using a Health in All Policies (HiAP) approach in the past two decades. It seeks to provide evidence on enabling factors that triggered three effective intersectoral public policies to improve population health in Mexico by improving nutritional, educational, and healthcare access conditions, reducing road traffic injuries, and addressing obesogenic feeding practices.

**Methods:**

We followed a qualitative approach to analyze the three intersectoral public policies selected as case studies. First, we designed an analytical framework to assess how intersectoral public policies are adopted, implemented, and sustained. The proposed framework is based on peer‐reviewed articles and gray public policy literature. Second, we used information from eleven semi‐structured interviews with key stakeholders conducted in previous research to identify more specific enablers and barriers of the three intersectoral policies selected according to predefined analytical categories used in the questionnaire.

**Results:**

The analysis showed three overall key findings. First, sound empirical evidence is essential for adopting a HiAP approach. Second, effective intersectoral mechanisms enhance implementation feasibility. Third, results‐based monitoring and evaluation contribute to the continuity of the analyzed intersectoral public policies. Finally, political support is needed throughout the policy process to maintain governance capacity and deliver results.

**Conclusion:**

We drew five global policy lessons that may be applicable in similar public policy settings in other countries. First, both technical and political enablers help set the intersectoral agenda. Second, effective communication is instrumental in convincing all stakeholders to address public health‐related policy issues. Third, political support at the highest level possible and the federal government's capacity are essential to implement sound policies. Fourth, several enablers exist for enhancing collaboration between ministries during implementation. Finally, monitoring and evaluation results are necessary for sustaining intersectoral policies beyond administrations.

## INTRODUCTION

After the Helsinki Statement on Health in All Policies (HiAP) was declared almost two decades ago [1], a renewed interest has analyzed how this approach has been implemented in several countries to improve population health [2, 3, 4, 5]. Back then, HiAP was defined as “an approach to public policies across sectors that systematically takes into account the health implications of decisions, seeks synergies, and avoids harmful health impacts to improve population health and health equity” [1](p2).

The more recent literature [3, 6] has shown how difficult joint policy‐making among different sectors can be because it requires setting common goals, delivering integrated responses, and providing increased accountability across government agencies. Furthermore, evidence on successfully implementing intersectoral policies is still limited [3, 6, 7], especially in low‐ and middle‐income countries, including Latin America [8]. Identifying factors enabling or hindering HiAP implementation has focused primarily on industrialized countries [4, 7], mainly at the local level [5]. Furthermore, the HiAP literature has analyzed either its policy dimensions [7, 9, 10] or political dimensions [11, 12, 13], except for a few exceptions that have followed a more integral approach [14, 15] as proposed here. This study aims to contribute to this lack of evidence by analyzing three national intersectoral public policy experiences in Mexico and identifying enablers and barriers throughout the HiAP adoption, implementation, and continuity process.

This study builds upon previous research that recognized the useful evidence of intersectoral actions in Mexico [16] for analyzing economic factors that enabled the adoption of intersectoral policies [17, 18]. We now focus on identifying and analyzing enabling factors that triggered three effective intersectoral public policies to improve population health in Mexico: the Education, Health and Nutrition Program (Progresa), the Mexican Road Safety Initiative (IMESEVI), and the National Agreement for Nutritious Health (ANASA). We also identified the main obstacles they faced to draw global lessons that may contribute to a better understanding of what factors hinder intersectoral public policy adoption, implementation, and continuity in other similar settings.

### Progresa

Progresa was a conditional cash transfer program created in 1997 that experienced two transformations, one in 2000 as Oportunidades and the other in 2013 as Prospera. Despite its long duration, it was terminated in 2019 for ideological reasons, given that its conditional nature was considered neoliberal and targeted [19]. Throughout these years, its objective was the same: To help households, in the long run, break out of a vicious cycle whereby poverty is transmitted from one generation to another. To achieve this, it provided financial incentives through cash transfers to promote child health, nutrition, and schooling.

### IMESEVI

IMESEVI was an intersectoral strategy initially headed by the Ministry of Health (MOH) in 2008 with the joint participation of the Ministry of Communications and Transportation, the Ministry of Public Security, and members of civil society. It sought to reduce road injuries, disability, and deaths by promoting road safety, preventing injuries, and improving care for the injured population [20]. Although the Ministry no longer heads the strategy, it is currently coordinated by a nonprofit organization as part of the Second Decade for Action for Road Safety, 2021–2030, supported by WHO.

### ANSA

ANSA was implemented in 2010 as an intersectoral strategy to promote healthy food and beverage consumption in every elementary and middle school across Mexico. Although 15 public government agencies and 5 business groups initially signed the agreement, including the MOH and Education, they failed to reach common public health objectives with other Ministries. The Ministry of Economy and the Ministry of Agriculture (MOA) continued to view regulation of the school environment as unnecessary and a potential threat to the food market and industry revenues [21]. In 2013, it became a component of a national strategy against overweight, obesity, and diabetes. Ten years later, it was legally strengthened with a new bill passed in Congress.

We selected these three intersectoral health public policies not only because they initially adopted an intersectoral approach based on sound evidence but also because all three were implemented for at least a decade beyond one national‐level administration.

## METHODS

**FIGURE 1:**
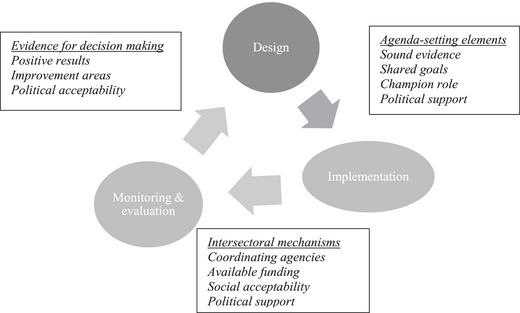
Framework for analyzing factors driving intersectoral public policies.

In several steps, we followed a qualitative approach to analyze these intersectoral health public policies selected as case studies. First, we conducted a literature review to identify concepts and categories for analyzing how intersectoral public policies are adopted, implemented, and sustained. We examined analytical approaches from three different but complementary perspectives: peer‐reviewed articles and gray public policy literature [10, 21–26], more recent and focused literature on intersectoral approaches [1, 2, 3, 4, 5, 6, 7, 8, 9], and political analysis of health policy [11, 12, 13, 14, 15]. These concepts and categories were then used to design the analytical framework shown in Figure 1.

### Analytical framework

The iterative intersectoral health public policy cycle is drawn mainly from the public policy literature [10, 21–26]. The specific enablers classified into three categories (agenda‐setting, intersectoral mechanisms, and evidence for decision‐making) were identified for each public policy phase (adoption, implementation, and evaluation) from the specific literature on the HiAP intersectoral approach [1, 2, 3, 4, 5, 6, 7, 8, 9]. To complement this public policy perspective, relevant analytical concepts were adopted from the politics of health policy literature [11, 12, 13, 14].

As Figure 1 shows, it suggests that there are three phases as part of an iterative intersectoral health public policy cycle. In its adoption or design phase, two critical analytical elements were identified. Initially, the design of public policies should be based on empirical evidence showing their technical feasibility in addressing a policy issue. This evidence may include either a situation diagnosis, a best practice to tackle a similar public health problem, or economic arguments such as the cost‐effectiveness of implementing a healthy public policy [16]. Sharing a vision that includes a joint agenda for intersectoral action, a legal mandate to enable enforcement, and leadership from the MOH drives an intersectoral public policy to its adoption [27].

During the implementation phase, all participating government agencies should align their goals by defining common objectives, maximizing budgets, and monitoring joint performance using shared indicators [27]. Additionally, acceptability among stakeholders is essential as well [11]. The cycle enters the third phase when the monitoring and evaluation results provide enough evidence for policy‐makers to decide whether they keep implementing the public policy as initially designed, scale it up, improve it if the results are positive, or discard it if it produces undesirable or unsatisfactory results. Furthermore, even though these are evidence‐based decisions, they might also be influenced by political factors [11].

### Data sources

Third, we used information from eleven semi‐structured interviews conducted in previous research [16]. Key informants with at least 10 years of experience making or studying health public policies in Mexico, representing academics and different public sectors, were interviewed in Spanish. A questionnaire was developed to identify specific enablers and barriers of each of the three intersectoral health public policies selected. The three basic analytical categories of the proposed framework above guided the content of the interviews: agenda‐setting elements for the adoption or design phase, intersectoral mechanisms for the implementation phase, and evidence for decision‐making for the monitoring and evaluation phase. Interviews were conducted between May and November 2012. Fourth, a review of the published gray and scholarly literature on the current policy process was undertaken to update the current situation of each intersectoral health public policy.

### Data analysis

Finally, to support study rigor, we triangulated data from several sources from each health public policy, from its creation to its current situation, including scholarly and gray literature and the key informants interviewed. Findings were assessed as adequate when evidence was from at least two data sources, for example, when comparing literature versus key informants or key informants representing different backgrounds or perspectives. We organized our results according to the proposed framework categories. Each policy phase had three broad categories: agenda‐setting elements, intersectoral mechanisms, and monitoring and evaluation. We then identified an enabler or obstacle for each category.

## RESULTS

Five senior researchers and six policymakers with at least 10 years of experience in public health, social development, and social security institutions were interviewed, as shown in Table 1. They represented diverse backgrounds, including physicians, economists, and health policy experts.

**TABLE 1:** Profile of key informants.

No	Affiliated institution	Relevant intersectoral experience
1	National Council for Evaluation of Social Policies	Coordinator and regulator of federal social policy evaluations
2	National Coordination of Oportunidades Program	Major role in the design and implementation of the Oportunidades Program for Human Development
3	Ministry of Health	Deputy Minister of Health for Health Prevention and Promotion
4	National Women's Institute	Major role in planning and evaluation of equity‐focused public policies
5	Research Institute for Economic Development	Senior economist and expert on inequality and poverty evaluation
6	National Commission for Social Protection on Health	Major role in the design and implementation of public health policies
7	Mexican Institute for Social Security	Senior economist and head of the financial unit
8	Harvard Kennedy School of Government	Major role in the evaluation of public policies designed and implemented by the Ministry of Social Development
9	Inter‐American Development Bank	Senior economist on social protection
10	National Institute of Public Health	Senior medical researcher on nutrition and public policy evaluation
11	National Institute of Public Health	Senior medical researcher on global health and social determinants of health

Table 2 summarizes the key factors that enabled or hindered the design or adoption, implementation, and continuity of the three intersectoral public policies analyzed.

**TABLE 2:** Key enablers and obstacles during the three policy stages.

	Prospera		IMESEVI	
	(1997–2019)		(2013–2023)	
	Enablers	Obstacles	Enablers	Obstacles
Design/Adoption	Sound evidence‐based design Political Presidential support Financial support from Ministry of Finance Aligned budgets, goals, and indicators from three ministries (Social Development, Education, and Health) Legal mandate		Strong evidence of health and economic impact of injuries High priority in the policy agenda Leadership role of MOH Shared agenda for reducing injuries, disability, and deaths Participation of NGOs Legal mandate signed by MOH, MOC, and state and local governments International financial support from Bloomberg Technical assistance from PAHO/WHO	
Implementation	Intersectoral Technical Committee for M&E Rules of operation shared by MOSD, MOE, MOH	Changing administrations and political priorities Funding cuts despite positive evaluation results	Sustainable support from international organizations through the Decades for Action for Road Safety agenda Monitoring at the national level	Allocation of insufficient financial resources Financial sustainability Reliable information to monitor performance at the state and local level Medical background from top decision‐makers from the Ministry of Health
Continuity	Results from M&E for operational feedback, policy improvement, and positive effects	Ended in 2019 for ideological and political reasons	Results from M&E for operational feedback, policy improvement and positive effects Alignment to SDGs Support from international and national NGOs	Financial feasibility MOH changing staff and loss of champions Lack of economic incentives for good performance

	ANSA			
	(2013–2023)			
	Enablers	Obstacles		
Design/Adoption	Sound empirical evidence of health and economic burden of overweight and obesity Legal mandate Leadership role of MOH	Regulation of food and beverages in schools, seen as a potential threat to the food market and industry revenues		
Implementation	Changes in policy strategy for enhancing feasibility (from school regulation to food and beverage taxes and labeling)	Financial sustainability Opposition from MOE and MOA Lack of enforcement rules at the school level Medical background from top decision‐makers from the MOH		
Continuity	Results from M&E for operational feedback, policy improvement Overweight and obesity remained a top public health issue Redesign and change of strategy to deliver better results	Opposition and strong lobbying from the unhealthy food and beverage industry Evaluations are not mandatory		

Abbreviations: M&E, monitoring and evaluation; MOA, Ministry of Agriculture; MOE, Ministry of Economy; MOE, Ministry of Education; MOF, Ministry of Finance; MOH, Ministry of Health; MOSD, Ministry of Social Development; SDGs, Sustainable Development Goals.

The following findings identify common enablers or obstacles to at least two analyzed public health policies. They are presented according to the three broad analytical categories of the framework that, in turn, correspond to each of the policy phases of design or adoption, implementation, and monitoring and evaluation for decision‐making.

### Agenda‐setting elements for adopting a HiAP approach

Sound empirical evidence sets the agenda for all three public intersectoral health policies. Prospera was initially designed based on strong empirical evidence. Nearly two decades before this, a growing body of scientific literature from the social and health sciences led to a more comprehensive understanding of poverty determinants [28]. This research emphasized the links between food intake, nutrition, health, and education, suggesting that an integrated rather than isolated approach to delivering services could effectively and efficiently tackle the low‐income population's nutritional, health, and educational needs. The accumulated empirical evidence helped to gradually persuade the Cabinet members to adjust the existing food subsidy and related poverty programs that led to the design of Progresa more than 20 years ago [28]. As a high‐level public health decision‐maker stated:

Health is an issue that should be addressed from a multisectoral perspective…if this needs to be better understood, it will be challenging to tackle most causes of health problems, except for genetic or biologically determined factors.

IMESEVI was adopted in the agenda in 2008 after a study gathered evidence showing that the isolated efforts of multiple institutions and agencies could not effectively address this multisectoral public health problem. Besides this intersectoral approach to reducing road traffic injuries, sound empirical evidence was crucial for advancing this initiative and identifying specific policy solutions. According to WHO, nearly half of the total road traffic deaths worldwide are related to the three most vulnerable road users: pedestrians, bicyclists, and motorcyclists. In Mexico, these users represent approximately 60% of all road traffic deaths [29]. Furthermore, the high economic burden of injuries was a compelling argument for including this initiative in the policy agenda. Together, road traffic‐related injuries, disabilities, and deaths account for nearly US$1 billion annually in Mexico [30].

Empirical solid evidence of overweight and obesity in Mexico and its economic consequences also made a significant contribution to set it high on the Federal Government agenda. The estimated direct cost of medical care attributable to overweight and obesity increased 61% from 26,283 million pesos in 2000 to approximately 42,246 million pesos in 2008. This represented 33% of the total medical budget available for that year. In 2008, the cost for 2017 was estimated at 77 919 million pesos [31]. On the other hand, the estimated indirect costs due to the productivity loss of premature death attributable to being overweight and obese have increased at an annual rate of 13.5%. Indirect and direct costs will increase to more than 150 million pesos by 2017. The economic burden these costs represent for the sustainability of the public healthcare system helped the MOH convince other sectors, particularly the Ministry of Finance, that intersectoral actions were urgently needed to address this public health problem. This evidence led to the design of ANSA, which included general guidelines for food and beverage consumption as one of its main strategies in every elementary and middle school nationwide.

MOH leadership was another crucial enabler for IMESEVI and ANSA. The MOH framed the issue in both cases to persuade other cabinet members. Furthermore, interviews evidence identified health and non‐health policymakers that health is a “politically correct” issue. Inside and outside the government, a health issue rarely faces opposition, except when it threatens the interests of a stakeholder such as the sweetened beverage industry. Health is considered socially acceptable because it contributes to an individual's well‐being, happiness, or satisfaction. As a top policy‐maker from the Ministry of Social Development stated:

What ultimately allowed us to sit at the same table, commit to a specific action, such as investing in sewage and improving housing conditions, and agree on a common goal was health improvement.

Legal mandates and high‐level political support were fundamental in articulating actions among participating government agencies. All three policy cases analyzed had a legal mandate. An initiative with strong political Presidential support and financial support from the Ministry of Finance legally mandated Progresa in 1997. Headed by the MOH, nearly 20 stakeholders signed the ANSA agreement in 2010, including fifteen public health agencies, whereas a deal signed by the MOH and the Ministry of Communications and Transportation in 2011 legally mandated IMESEVI, with the participation of both state and local governments.

Political will and decision‐making at the highest level were crucial for prioritizing these public policies on the political agenda. All of the intersectoral public policies analyzed here had support from the ministers of the sectors involved, and, apart from IMESEVI, all received backing from the President.

Besides these legal and political mechanisms, all three intersectoral policies built a shared vision at its adoption phase by sharing common goals. With Prospera, the Ministry of Social Development, Health, and Education aligned their budgets, goals, and indicators. Representatives from the various public agencies shared the same goals of reducing traffic‐related deaths by 50% and diminishing injuries and disabilities [32]. At the same time, ANSA participants agreed on the common goal of reducing the burden of overweight and obesity. Furthermore, several interview decision‐maker respondents believed allocating specific resources to achieve common goals would create positive incentives for a synergistic effect. Shared goals were critical for effectively aligning all actors. A policy‐maker from the Ministry of Social Development stressed its importance:

To pursue the same goals, each sector must recognize that its actions contribute to achieving them. Identifying indicators to measure such goals was more challenging, and even if an indicator was agreed upon, reliable information was only sometimes available to measure it.

### Intersectoral effective mechanisms for implementation

Findings showed that implementation is feasible when adequate mechanisms are established to conduct an intersectoral approach effectively. One enabler is formal coordinating bodies or committees, such as permanent intersectoral task force groups, which have helped articulate actions across sectors. The Technical Committee of Prospera summons middle‐level officials from the ministries of social development, health, education, finance, and transparency to attend regular meetings where decisions are made regarding changes to the operational rules of the program; performance is assessed using key indicators and where evaluation results are reviewed. This required sectoral alignment to effectively coordinate the activities of the implementing agencies and ministries within the Executive Branch. Common goals were set for all of them. Budgets were reallocated to each of them to achieve such goals. Moreover, shared performance indicators were agreed upon. These shared intersectoral elements were published yearly in the so‐called operational rules and are regularly monitored by Congress and the SHCP. In the case of ANSA, when it was transformed into the Alliance for Foodborne Health in 2019, the Health, Nutrition, and Environment Intersectoral Group (GISAMAC) has helped to articulate food security intersectoral actions enabled coordination, engaged communication, and shared monitoring between four ministries—Health, Economy, Agriculture, and Environment.

Other shared intersectoral elements were identified in official documents and corroborated in the interviews in health policy areas of mutual interest, such as public elementary schools. These were the most feasible intersectoral policy spaces to ban unhealthy food for school‐aged children consumption. The MOH understood that, but more importantly, the Ministry of Education acknowledged the potential effects of preventing obesity and overweight in school‐aged children.

Financial and technical supports from national and international organizations have also enabled these intersectoral public policies. In the case of Prospera and ANSA, funding was provided by the Ministry of Financing. Technical assistance for Prospera came from the World Bank and the Inter‐American Development Bank, whereas the National Institute of Public Health headed the technical support for ANSA. On the other hand, few resources had been allocated to the MOH to prevent injuries, and little could be achieved. However, with the leadership of the National Center for Injury Prevention, the agency within the MOH in charge of these issues, together with international financial support from the Bloomberg Foundation and technical support from PAHO and WHO, this initiative, which started as a pilot study, soon became the National Strategy for Road Safety. WHO's call for the First (2010–2020) and the Second Decade for Action for Road Safety (2021–2030) has helped support this initiative. Furthermore, this strategy has been aligned with the 2030 Agenda for Sustainable Development, reiterating the goal of reducing by 50% the number of deaths from road traffic injuries by 2030 [33].

Obstacles during implementation have led to changes in the scope and approaches of these intersectoral public policies. The regulation component of ANSA should have been more effectively enforced in schools, given the vested interests of the food and beverage industry, despite substantial scientific evidence and increasing awareness of the population at risk [34]. However, overweight and obesity remained a significant public health problem. Therefore, in 2014, ANSA became a component of a more comprehensive national strategy to address this problem, including the regulation of food and beverage advertising targeted to children, taxes on sweetened beverages, food labeling of obesogenic products, and an information system to monitor and evaluate the intersectoral public policies [33]. The tax on sweetened beverages took effect in 2014 despite the opposition from the Ministry of Economy and the MOA, fearing job losses [34]. This fiscal strategy reduced consumption by up to 10% [35] (p2). More recently, the national strategy became the Alliance for Health Food. A new food labeling in 2020 targeted five ingredients in processed foods harmful to health—saturated and trans fats, calories, sugars, and sodium—to diminish their consumption [36]. Early findings show that people are aware of their harmful effects and have started to reduce their consumption [37].

According to some key informants, the health sector may hinder successful implementation. The MOH has historically adopted a medical‐care approach to address population health. Most of the top decision‐makers in the health sector have been physicians who need a public health background, preventing them from fully understanding social factors such as income, education, housing, or transportation as determinants of health. Most respondents acknowledged that this knowledge gap requires investment in training policy‐makers from within and outside the health sector, especially legislators who, besides the Federal Government, can decide how resources are allocated between the multiple public programs and policies in Mexico. They emphasized, however, that training should focus on medical and health professionals, who should assume a leadership role in fostering a HiAP approach.

### Evidence for decision‐making

Findings showed that monitoring and evaluation results provided evidence for policymakers to decide whether to keep implementing the public policy as initially designed, improve it, or change it if necessary. Results‐based evidence played a crucial role in the continuity of Prospera until 2019 when it was terminated. Since its design, as Progresa, it has been systematically evaluated by international and national independent academic researchers, who have measured its effects and identified windows of opportunity to improve its design [38]. Despite being canceled a few years ago, it still provides relevant evidence that HiAP is feasible and has significant positive equity and health effects.

Evaluations were also essential for ANSA to obtain operational feedback, propose improvements, and measure its impact [20]. Despite some promising evidence from evaluations that showed healthier food consumption from products available at schools [39, 40], this strategy did not show an overall positive impact. The percentage of overweight school‐aged children decreased from 20 in 2012 to 18.7 in 2021, but obesity grew from 14.7 to 18.7 in the same period [41].

However, not all public policies are rigorously evaluated, except for Prospera. Although every health‐related policy should undergo some form of evaluation by law, existing evaluation guidelines are only sometimes followed because they are difficult to enforce effectively. Moreover, although it is recommended that health‐related policies be subject to regular and rigorous evaluation before, during, and after implementation, there is a need for economic assessments in evaluations completed. This contributes to the unavailability of valuable and reliable information, which can become an obstacle for monitoring and evaluating implementation, according to one of the senior researchers interviewed:

The primary sources of good and reliable health information usually do not gather cost and monetary information. In contrast, excellent and reliable information on costs and monetary terms is not collected for health monitoring purposes.

The analysis and the interviews also suggest that international evidence and support from WHO are essential when seeking support from difficult stakeholders, such as industries producing food high in sugar, salt, and fat. Signing the ANSA agreement would not have been feasible without the call from WHO to reduce obesity and the available empirical evidence to support it. Similar backing was necessary for incorporating the prevention of road traffic injuries into the MOH's agenda with the IMESEVI initiative.

Even if the best evidence regarding health, equity, or economic impact were available, it would need to be translated into action, which can become an obstacle to continuity. To achieve this, two elements are essential, according to one of the top government officials interviewed:

First, it requires that decision‐makers clearly understand that addressing social determinants will ultimately impact health …and second, effective leadership that enforces a shared intersectoral agenda based on strong empirical evidence.

Although ANSA and IMESEVI still exist as intersectoral public policies, they have been redesigned to meet similar objectives but with different approaches. Ten years after it was implemented, IMESEVI has remained on the policy agenda with the leadership role of the National Alliance for Road Safety, a nongovernmental organization with an advisory and surveillance role to the Federal government. It maintained its HiAP approach, but the MOH no longer headed it [42].

On the other hand, ANSA, after being transformed into a national strategy to overcome overweight and obesity in 2014, became the Alliance for Food‐Born Health in 2019. Its most important component, a new food labeling in 2020, targeted five ingredients in processed foods harmful to health—saturated and trans fats, calories, sugars, and sodium—to diminish consumption [43]. Early findings show that people are aware of their harmful effects and have started to reduce their consumption [41].

## DISCUSSION

This study identified both enablers and barriers of HiAP adoption, implementation, and continuity based on analyzing three national‐level intersectoral public policies in Mexico that remained operational for at least a decade, considering their technical and political aspects, which are seldomly analyzed together, except for a few exceptions [14, 15], The three experiences from Mexico contribute to the literature on intersectoral approaches to health policy as additional case studies to better understand the design, implementation, and continuity processes of health policies that work across sectors.

For this purpose, we developed a more comprehensive analytical framework that integrated all policy phases [7–10, 22] and the political dimensions [11, 12, 13, 14] of analysis, suggested separately in most frameworks. This framework allowed for a more thorough analysis of the policy process by identifying enablers that make it more feasible to adopt and implement intersectoral public policies and those that keep them on the agenda and operating in Mexico and other countries with similar contexts. Furthermore, it provides evidence that a HiAP approach is viable despite the policy and political barriers also identified in the analyzed intersectoral public policies of a middle‐income country like Mexico.

Evidence from this analysis identified the same factors other studies found enabling the adoption of intersectoral policies. These included a sound evidence‐based design and a shared vision with common goals for setting public health issues in the agenda [27, 40]. Political factors were also found, including high‐level political support from the President and a legal mandate [10, 13]. As evidenced in other studies, MOH did not necessarily play a champion or leader role [27], with Prospera and IMESEVI in its current phase, this lack of leadership from the health sector, identified as an obstacle or challenge, could be attributed to the medical background of its top decision‐makers and its short‐sighted vision of nonmedical factors influencing health, as expressed by several key informants. The other obstacle consistent with the literature for adopting ANSA was opposition from stakeholders such as the MOA and the MOE within the government and the processed food and sweetened beverage industry that feared profit losses from taxes [14, 44] or regulation through labeling [13, 20].

Effective mechanisms for implementing intersectoral policies with a HiAP approach have been less studied [2, 3]. As shown in other studies [2, 3, 27], we found that committees such as the one used in Prospera for monitoring performance [41] or the GISAMAC for articulating food security intersectoral actions enabled coordination, engaged communication, and shared monitoring between four ministries [37]. Financial support or technical assistance from international organizations, a key enabler identified in the literature [5, 27], was also found in all three intersectoral public policies analyzed. Social acceptability from policymakers and beneficiaries of the intersectoral policies was an additional enabler found in the Mexican context [11, 13, 15, 34, 35, 44] that has been little explored in other studies, except when stakeholder analysis is conducted [39, 45].

Delivering results is a crucial enabler for the continuity of intersectoral public policies, as other studies have shown [46, 47, 48, 49] Monitoring and evaluation, as the analytical framework suggested, provide evidence to decide whether policymakers keep implementing the public policy as initially designed if the results are positive or adjust it, as was the case with Progresa changing its name with different administrations. Still, its original design remained essentially the same.

Our findings also showed that intersectoral public policies may remain and be transformed when the strategic policy issue is still relevant, as with overweight and obesity, with ANSA changing to the Alliance for Food‐Born Health and traffic injuries with IMESEVI to the National Alliance for Road Safety. ANSA changed its focus from a policy fostering healthy eating behavior in schools to a potentially higher impact agenda enforcing health taxes and labeling food and beverages [50]. IMESEVI lost its MOH champion but remained as a civil society initiative with international WHO support from the Second Decade for Action for Road Safety (2021–2030) through a National Alliance [31].

Finally, the evidence suggested that political support is critical for continuity, even if intersectoral public policies can improve health or achieve their intended goals [2, 11]. On the one hand, Prospera was eliminated for ideological reasons despite its strong evidence of improving its targeted population's health, nutrition, and education conditions. At the same time, ANSA and IMESEVI remained intersectoral public policies with weaker evidence of their positive effects on health [51, 52].

Triangulating academic and gray literature, including official government documents and interviews with key informants, contributed to a more rigorous analysis. However, as with any qualitative study, its limitations should be acknowledged. Although these findings provide useful information for better understanding what enabled and hindered the HiAP approach in three Mexican intersectoral public policies, as evidenced in other settings, these three experiences may need to be more generalizable to every HiAP approach.

More key informants would have allowed for a more in‐depth analysis. However, this limitation was addressed using the academic and gray literature. Reliability is another limitation, even though this study proposed a comprehensive framework that included policy and political factors and that can be replicated in similar settings in other countries. To spur further knowledge and value‐added in future research, we suggest identifying additional political contexts and processes of other policies in other settings and critical stakeholders for working effectively across sectors to address public health issues.

## CONCLUSIONS

Findings from this analysis contribute to identifying enablers and obstacles for adopting, implementing, and sustaining intersectoral health public policies by analyzing three Mexican experiences. The proposed analytical framework helped better understand these policy processes by incorporating a political analysis perspective. Furthermore, it may provide lessons learned to inform future intersectoral efforts in similar policy and political contexts. First, sound evidence‐based design, aligned goals, and a legal mandate help set an intersectoral agenda. However, more technical enablers are needed than these. The main obstacles faced in adopting intersectoral public policies are stakeholders that oppose them, given their vested interests. Thus, political support at the highest level of government is essential to push forward the agenda, but a champion, such as the MOH, is also needed to drive implementation. Lack of sufficient financial resources to adopt and implement intersectoral public policies like those analyzed may become another obstacle.

Additionally, several enablers exist for enhancing collaboration between ministries during implementation, such as committees, joint monitoring, aligned budgets, and acceptability from all or most stakeholders. Finally, monitoring and evaluation results are necessary, but more is needed for decision‐making regarding policy changes to improve their effectiveness and sustain intersectoral policies beyond administrations. They need to be more enablers for their continuity. Political support from critical stakeholders is essential for the continuity of intersectoral public policies with health dimensions.

## AUTHOR CONTRIBUTIONS

Adolfo Martinez‐Valle conceptualized, designed, and wrote the paper. Alejandro Figueroa‐Lara revised the manuscript and provided inputs. Adolfo Martinez‐Valle and Alejandro Figueroa‐Lara conducted the interviews.

## CONFLICT OF INTEREST STATEMENT

Adolfo Martinez Valle was director general of planning and evaluation of the Oportunidades program, one of the public policies analyzed, from 2009 to 2011.

## Data Availability

Data from the interviews and the questionnaire are available from the main author if requested.
